# Long-term trends in the loss in expectation of life after a diagnosis of chronic lymphocytic leukemia: a population-based study in the Netherlands, 1989–2018

**DOI:** 10.1038/s41408-022-00669-7

**Published:** 2022-04-20

**Authors:** Lina van der Straten, Carolien C. H. M. Maas, Mark-David Levin, Otto Visser, Eduardus F. M. Posthuma, Jeanette K. Doorduijn, Anton W. Langerak, Arnon P. Kater, Avinash G. Dinmohamed

**Affiliations:** 1 Department of Research and Development, Netherlands Comprehensive Cancer Organisation (IKNL), Utrecht, The Netherlands; 2 Department of Internal Medicine, Albert Schweitzer Hospital, Dordrecht, The Netherlands; 3 Laboratory Medical Immunology, Department of Immunology, Erasmus MC, Rotterdam, The Netherlands; 4 Department of Public Health, Erasmus University Medical Center, Rotterdam, The Netherlands; 5 Department of Registration, Netherlands Comprehensive Cancer Organisation (IKNL), Utrecht, The Netherlands; 6 Department of Internal Medicine, Reinier The Graaf Hospital, Delft, The Netherlands; 7 Department of Hematology, Leiden University Medical Center, Leiden, The Netherlands; 8 Erasmus MC Cancer Institute, Department of Hematology, University Medical Center Rotterdam, Rotterdam, The Netherlands; 9 Amsterdam UMC, University of Amsterdam, Department of Hematology, Cancer Center Amsterdam, Lymphoma and Myeloma Center Amsterdam, Amsterdam, The Netherlands; 10 Amsterdam UMC, Vrije Universiteit Amsterdam, Department of Hematology, Cancer Center Amsterdam, Amsterdam, The Netherlands

**Keywords:** Chronic lymphocytic leukaemia, Epidemiology

## Dear Editor,

We read with interest the article by Kajüter and colleagues about the relative survival of 2,327 patients with chronic lymphocytic leukemia (CLL) diagnosed from 1993 to 2016 in Münster, Germany [1]. Their study assessed the impact of the introduction of chemoimmunotherapy for CLL management based on 5-year relative survival. Congruent with our recent population-based findings among 20,468 CLL patients diagnosed between 1989 and 2016 in the Netherlands [2], 5-year relative survival in CLL increased markedly since the introduction of chemoimmunotherapy, approximating 90% for patients up to age 70 [1, 2].

Relative survival is a frequently used measure in population-based cancer research to assess progress in patient management over time. Seeing the comparatively favorable prognosis of CLL patients in modern times, other survival measures may be more informative because relative survival does not inform on patient survival across the entire remaining life span.

The application of the loss in expectation of life (LEL) has recently entered the arena of population-based cancer research to assess the impact of a cancer diagnosis on the patients’ life expectancy and the average number of life-years lost [3]. As the LEL has not been assessed for CLL patients, our nationwide, population-based study complements and extends the study of Kajüter and colleagues by estimating the life expectancy of CLL patients.

We selected CLL patients diagnosed between 1989 and 2018—with survival follow-up through December 31, 2020—from the nationwide Netherlands Cancer Registry (NCR) using the International Classification of Diseases for Oncology morphology code 9823. The NCR includes all newly diagnosed malignancies in the Netherlands since 1989, with nationwide coverage of at least 95%. Further details about the registry are published elsewhere [2]. Patients’ survival was followed from the date of diagnosis to death, emigration, or end of follow-up, whichever occurred first. Seventy-one patients diagnosed at autopsy were excluded. According to the Central Committee on Research involving Human Subjects (CCMO), this type of observational study does not require approval from an ethics committee in the Netherlands. The Privacy Review Board of the NCR approved the use of anonymous data for this study.

We report four statistical measures to evaluate life expectancy. The first measure is LEL, quantifying the difference in life expectancy between patients and the general population, of which the latter is matched to the patients by age, sex, and calendar year. Here, the LEL is interpreted as the average number of life-years lost due to a CLL diagnosis. The LEL can vary markedly across ages because life expectancy is age-dependent. Therefore, the proportional LEL (PLEL) was estimated as a second measure to assess the prognostic effect of age on survival. The PLEL was calculated as the LEL divided by the population life expectancy. Since excess mortality may diminish with each additional year survived post-diagnosis, we estimated the LEL conditional on surviving each additional year up to ten years post-diagnosis (i.e., conditional LEL; CLEL). Lastly, the CLEL was also corrected for the prognostic effect of age, yielding the proportional conditional LEL (PCLEL) as a fourth measure.

The survival measures were modeled using restricted cubic splines within the framework of a flexible parametric relative survival model [4]. The sex-specific survival measures were presented by year of diagnosis for four age categories at diagnosis (i.e., 50, 60, 70, and 80 years), unless otherwise stated. Details about the statistical modeling are provided in the Supplemental Methods. All analyses were performed using Stata/SE version 17.0 (StataCorp, TX, USA).

**Fig. 1:**
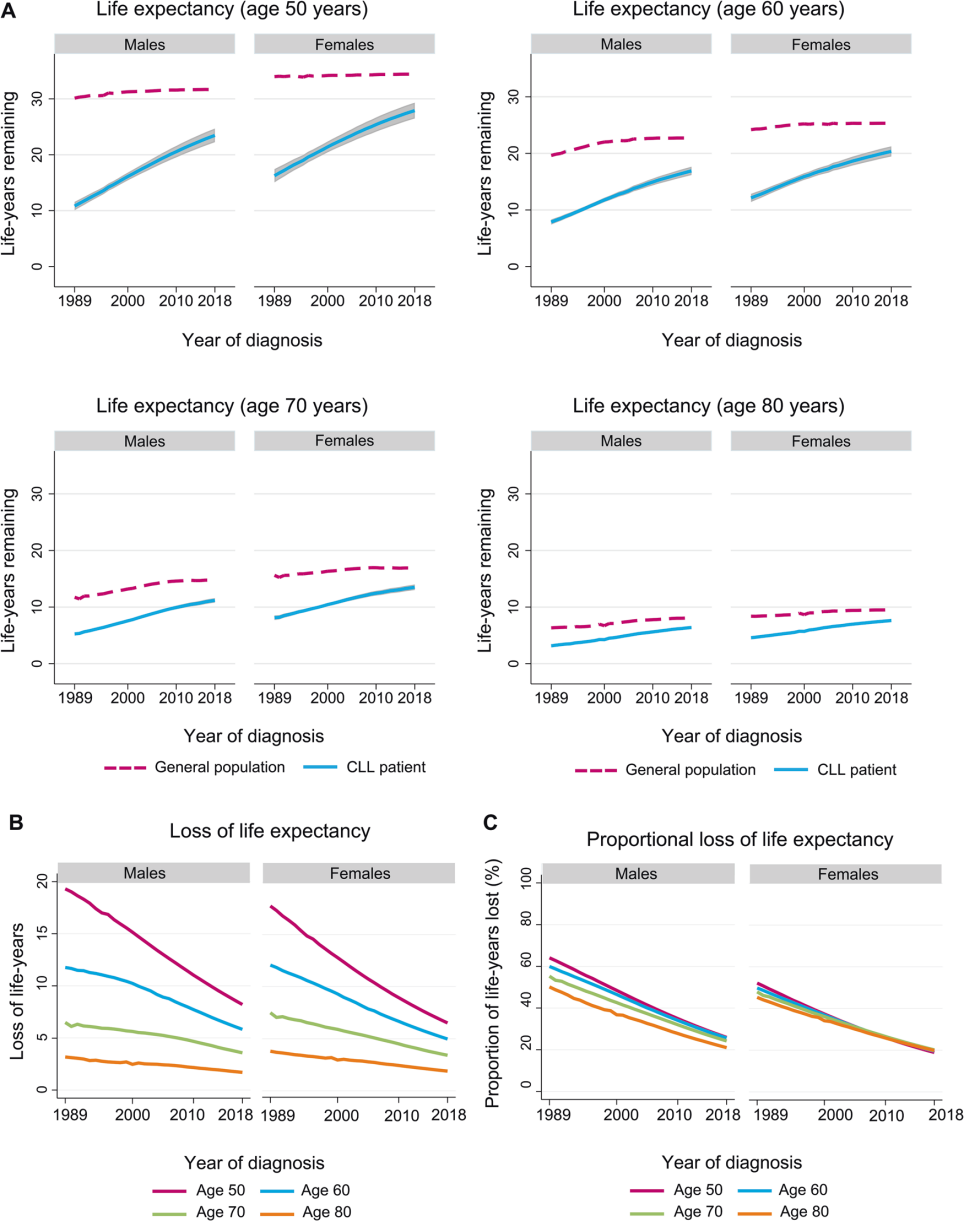
Trends in various life expectancy measures of patients with chronic lymphocytic leukemia diagnosed in the Netherlands between 1989 and 2018. Panel **A** depicts the life expectancy of the general population (dashed lines) and patients with chronic lymphocytic leukemia (solid lines) by year of diagnosis for four age categories, stratified by sex. The shaded area around the life expectancy of patients with chronic lymphocytic leukemia portrays the 95% confidence interval for the point estimates, which was obtained using the Delta method. Panel **B** presents the loss in expectation of life (LEL) of patients with chronic lymphocytic leukemia by year of diagnosis for four ages, stratified by sex. Panel **C** presents the proportional loss in expectation of life (PLEL) of patients with chronic lymphocytic leukemia by year of diagnosis for four ages, stratified by sex. The projected measures of life expectancy according to selected years of diagnosis are presented in Supplementary Table 2. Abbreviation: CLL, chronic lymphocytic leukemia.

Our analytic cohort included 23,692 CLL patients (median age, 69 years; interquartile range, 61–77 years; 61% males) diagnosed in the Netherlands between 1989 and 2018 (Table S1). The life expectancy of CLL patients increased for all four age categories between 1989 and 2018 (Fig. 1A). This absolute increase was most pronounced for patients aged 50 and 60 years at diagnosis. For example, a 50-year-old male diagnosed with CLL in 1990 and 2018 would have 11.3 (95% confidence interval [CI], 10.6–12.0) and 23.5 (95% CI, 22.3–24.7) life-years remaining, respectively (Fig. 1A and Table S2). On the other hand, an 80-year-old male diagnosed with CLL in 1990 and 2018 would have 3.3 (95% CI, 3.1–3.5) and 6.4 (95% CI, 6.2–6.6) life-years remaining, respectively (Fig. 1A and Table S2).

The increase in the life expectancy of CLL patients was greater than in the general population. Consequently, the LEL (Fig. 1B) and PLEL (Fig. 1C) of CLL patients across all four age categories decreased over time. The decrease in LEL was most pronounced in patients aged 50 and 60 years at diagnosis (Fig. 1B). Despite the decreasing LEL, all studied age groups diagnosed in 2018 had excess mortality, reflected in an LEL ranging from 1.7 to 8.2 years, depending on age and sex (Fig. 1B and Table S2). Of note, the decrease in LEL was markedly less in older patients (i.e., 70 and 80 years) since elderly individuals generally have fewer life-years remaining than younger individuals (Fig. 1B and Table S2). Indeed, estimates of PLEL showed that the age differential in survival became less pronounced over time and eventually dissipated for female patients (Fig. 1C and Table S2). The PLEL was ~20% for both sexes across all studied age groups in 2018, indicating excess mortality akin to findings from the LEL (Fig. 1C and Table S2).

**Fig. 2:**
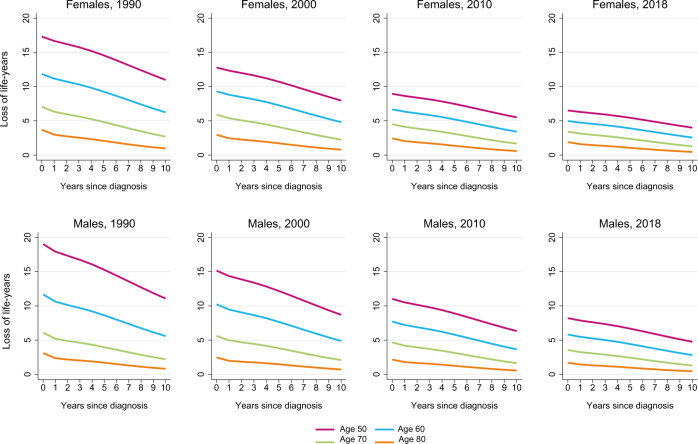
The conditional loss in expectation of life of patients with chronic lymphocytic leukemia diagnosed in the Netherlands. The conditional loss in expectation of life (CLEL) is presented according to four age categories at diagnosis, stratified by sex, and four selected calendar periods of diagnosis. The projected measures of life expectancy according to age categories are presented in Supplementary Table 3.

Overall, the CLEL decreased with each additional year survived post-diagnosis, irrespective of age and sex (Fig. 2). For patients diagnosed in 1990, there was a substantial decrease in the CLEL with additional years survived post-diagnosis. In more recent years, the slope of the CLEL became less steep since the LEL in these years is already comparatively low. Nevertheless, CLL patients diagnosed in 2018 who survived up to ten years post-diagnosis still lost 0.5 to 1.0 life-years, depending on age and sex (Table S3). The PCLEL estimates demonstrated that (i) the age differential in the PCLEL became less conspicuous over time and (ii) excess mortality persisted for contemporary diagnosed patients, irrespective of age and sex (Fig. S1 and Table S4).

This nationwide, population-based study demonstrates that the life-years lost decreased in CLL patients diagnosed in the Netherlands between 1989 and 2018, regardless of age and sex. This study, which is the first of its kind, complements and extends the findings by Kajüter and colleagues [1] because we went beyond survival up to 5 years post-diagnosis by estimating the longevity across the entire patients’ life span.

The steady progress in CLL management across several treatment lines over the past decades is a credible factor contributing to the continuous increase in the life expectancy of CLL patients. The improvement in life expectancy during the 1990s and early 2000s may be attributed to the broader application of purine analogs in combination with alkylating agents, particularly fludarabine-cyclophosphamide [5, 6]. Between the mid-2000s and early-2010s, the addition of rituximab to chemotherapy heralded a new era for CLL management, most likely resulting in the increased life expectancy as of the mid-2000s. Indeed, the pivotal CLL8 and CLL11 studies showed a marked improvement in progression-free survival (PFS) and overall survival (OS) in patients treated with first-line chemoimmunotherapy, as compared to chemotherapy alone, in medically fit and unfit patients, respectively [7, 8]. More recently, the chemoimmunotherapy paradigm has shifted towards more novel targeted agents (e.g., ibrutinib and venetoclax). These agents exert the capacity to improve PFS dramatically across various therapy lines [9–13]. Also, the combination of ibrutinib-rituximab improves OS compared to conventional chemoimmunotherapy [9]. However, these novel targeted approaches have been available in the Netherlands from 2014 onwards for selected patient populations. Therefore, it is premature to conclude on the impact of these novel approaches on life expectancy. We thus encourage monitoring the progress in the population level survival of CLL patients since excess mortality persists in modern times, even for CLL patients surviving up to ten years post-diagnosis [14]. Of note, earlier detection of CLL might have artificially influenced the life expectancy measures. However, this would only potentially influence estimates for patients diagnosed during the 1990s since the age-standardized incidence rate of CLL in the Netherlands remained comparatively steady as of the early 2000s, and the life expectancy of CLL patients continued to increase thereafter [2].

The strengths of our study include the use of population-based data from a long-running and well-established cancer registry. As such, we could estimate life expectancy from historical and contemporary perspectives. Limitations of our study encompass the lack of detailed patient and CLL characteristics, such as socioeconomic status and Rai stage, to further stratify life expectancy according to these baseline characteristics. Also, LEL estimates for more recently diagnosed patients rely on extrapolation. Then again, extrapolation was reasonably accurate in previous studies with comparatively short survival follow-up [15]. Nevertheless, the estimates in recent years might be underestimated in light of recent progress with novel therapeutic approaches.

In summary, the life expectancy of CLL patients diagnosed between 1989 and 2018 increased steadily in the Netherlands. This increase is likely attributed to the broader application of more efficacious therapies over time. Notwithstanding, continuous population-based surveillance is essential to assess the impact of the rapidly evolving management of CLL on survival since excess mortality is still a threat in contemporary diagnosed and managed patients.

## Supplementary information

Online Appendix
