# Supplementary material for: Long-term trends in the loss in expectation of life after a diagnosis of chronic lymphocytic leukemia: a population-based study in the Netherlands, 1989–2018
Source: Blood Cancer J. 2022 Apr 20;12(4):72. doi: 10.1038/s41408-022-00669-7 (PMC9021189; doi:10.1038/s41408-022-00669-7)
Supplement: Supplementary file 1 — Online Appendix [file 41408_2022_669_MOESM1_ESM.pdf]

ONLINE APPENDIX

Title

Long-term trends in the loss in expectation of life after a diagnosis of chronic lymphocytic leukemia: a population-based study in the Netherlands, 1989-2018

Short title

The life expectancy of CLL patients

Authors and affiliation

Lina van der Straten,[1-3,*] Carolien C.H.M. Maas,[1,4,*] Mark-David Levin,[2] Otto Visser,[5] Eduardus F.M. Posthuma,[6,7] Jeanette K. Doorduijn,[8] Anton W. Langerak[3], Arnon P. Kater,[9] Avinash G. Dinmohamed[1,4,9,10]

[1]Department of Research and Development, Netherlands Comprehensive Cancer Organisation (IKNL), Utrecht, The Netherlands; [2]Department of Internal Medicine, Albert Schweitzer Hospital, Dordrecht, The Netherlands; [3]Laboratory Medical Immunology, Department of Immunology, Erasmus MC, Rotterdam, The Netherlands; [4]Erasmus MC, Department of Public Health, University Medical Center Rotterdam, Rotterdam, The Netherlands; [5]Department of Registration, Netherlands Comprehensive Cancer Organisation (IKNL), Utrecht, The Netherlands; [6]Department of Internal Medicine, Reinier The Graaf Hospital, Delft, The Netherlands; [7]Department of Hematology, Leiden University Medical Center, Leiden, The Netherlands; [8]Erasmus MC Cancer Institute, Department of Hematology, University Medical Center Rotterdam, Rotterdam, The Netherlands; [9]Amsterdam UMC, University of Amsterdam, Department of Hematology, Cancer Center Amsterdam, Lymphoma and Myeloma Center Amsterdam, Amsterdam, The Netherlands; [10]Amsterdam UMC, Vrije Universiteit Amsterdam, Department of Hematology, Cancer Center Amsterdam, Amsterdam, The Netherlands; [*]shared first-authorship

Supplemental methods

The flexible parametric survival model estimates the life expectancy of patients with chronic lymphocytic leukemia (CLL) using several parameters that require tuning (1). We started with a model with (i) two degrees of freedom to model the baseline hazard and age at diagnosis, (ii) one degree of freedom to model year at diagnosis, and (iii) zero degrees of freedom to model time-dependent effects. The time of cure was set at twenty years post-diagnosis because we expect excess mortality to persist for at least 20 years for CLL patients based on our prior research (2).

Next, we investigated whether our initial model could be improved by adding flexibility to the model. This step was undertaken to more accurately predict the survival curve of CLL patients without creating issues related to convergence due to sparse observations in specific patient groups based on the combination of the year of diagnosis, age at diagnosis, and sex. The improvement of our initial model was achieved by changing the degrees of freedom of one parameter while keeping the remaining parameters constant. First, the baseline hazard was allowed to vary from one to ten while keeping the degrees of freedom of the remaining parameters constant as per the settings of the initial model. The number of degrees of freedom to model the baseline hazard is chosen based on the model with the smallest BIC. Second, the number of degrees of freedom to model age at diagnosis varied from one to three, with the baseline hazard modelled with the number of degrees of freedom determined from the previous iteration, while setting the number of degrees of freedom of the other parameters as per the initial model. Thirdly, the number of degrees of freedom to model year at diagnosis varied from one to three, using the parameters from the previous two steps and the degrees of freedom to model time-dependent effects as defined in the initial model. Lastly, the time-dependent effects varied from zero to two, with all the other parameters set to the optimal choice resulting from the previous steps.

The final, most optimal model parameters for CLL patients are set at (i) five degrees of freedom to model the baseline hazard, (ii) two degrees of freedom to model age at diagnosis, (iii) one

degree of freedom to model calendar year at diagnosis, and (iv) one degree of freedom to model time-dependent effects.

Supplemental Tables

Supplemental Table S1. Demographic characteristics of patients with chronic lymphocytic leukemia in the Netherlands, 1989-2018.

	**Total**		**1989 – 2000**		**2001 - 2010**		**2011 – 2018**	
**Characteristics**	**No.**	**%**	**No.**	**%**	**No.**	**%**	**No.**	**%**
**Total patients**	23,692	100	6,522	28	8,553	36	8,617	36
**Age [median; IQR]**	69	[61; 77]	70	[62; 77]	69	[61; 77]	69	[62; 77]
18-49	1,287	5	373	6	501	6	413	5
50-59	3,620	15	940	14	1,418	17	1,262	15
60-69	7,048	30	1,842	28	2,549	30	2,657	31
70-79	7,581	32	2,195	34	2,633	31	2,753	32
80-97	4,156	18	1,172	18	1,452	17	1,532	18
**Males**	14,392	61	3,846	59	5,186	61	5,359	62
**Deaths**	14,061	59	5,973	92	5,579	65	2,509	29

Abbreviation: IQR, interquartile range

Supplemental Table S2. The life expectancy of the general population and patients with chronic lymphocytic leukemia, the loss in expectation of life of patients with chronic lymphocytic leukemia, and the proportional loss in expectation of life of patients with chronic lymphocytic leukemia. These survival measures, with associated 95% confidence intervals, are presented for four selected calendar years and ages at diagnosis.

Measure	Age 50 years				Age 60 years				Age 70 years				Age 80 years			
	Males		Females		Males		Females		Males		Females		Males		Females	
**1990**																
LE	30.3		34.1		19.9		24.3		11.4		15.2		6.4		8.4	
LE CLL	11.3	(10.6; 12.0)	16.7	(15.6; 17.9)	8.2	(7.8; 8.6)	12.5	(11.8; 13.2)	5.3	(5.1; 5.6)	8.2	(7.8; 8.6)	3.3	(3.1; 3.5)	4.7	(4.4; 4.9)
LEL	19.0	(18.3; 19.8)	17.3	(16.2; 18.4)	11.7	(11.2; 12.1)	11.8	(11.1; 12.5)	6.1	(5.8; 6.4)	7.0	(6.7; 7.4)	3.1	(2.9; 3.3)	3.7	(3.5; 3.9)
PLEL (%)	62.7	(60.4; 65.1)	50.8	(47.5; 54.1)	58.7	(56.6; 60.9)	48.7	(45.9; 51.5)	53.3	(51.1; 55.5)	46.2	(43.8; 48.7)	48.7	(45.7; 51.7)	44.2	(41.3; 47.0)
**2000**																
LE	31.3		34.2		22.0		25.2		13.2		16.3		6.7		8.7	
LE CLL	16.1	(15.4; 16.8)	21.4	(20.4; 22.4)	11.8	(11.4; 12.2)	15.9	(15.3; 16.5)	7.6	(7.4; 7.8)	10.4	(10.1; 10.7)	4.2	(4.1; 4.4)	5.7	(5.5; 5.9)
LEL	15.2	(14.5; 15.8)	12.8	(11.8; 13.8)	10.2	(9.9; 10.6)	9.3	(8.7; 9.9)	5.6	(5.4; 5.8)	5.9	(5.6; 6.2)	2.5	(2.3; 2.6)	3.0	(2.8; 3.1)
PLEL (%)	48.5	(46.3; 50.7)	37.4	(34.6; 40.2)	46.5	(44.8; 48.3)	36.9	(34.6; 39.2)	42.6	(41.1; 44.2)	36.1	(34.3; 37.9)	36.8	(34.8; 38.8)	34.1	(32.2; 36.1)
**2010**																
LE	31.6		34.3		22.7		25.3		14.6		17.0		7.8		9.4	
LE CLL	20.5	(19.5; 21.6)	25.4	(24.1; 26.7)	14.9	(14.3; 15.5)	18.6	(17.9; 19.4)	9.9	(9.6; 10.2)	12.5	(12.0; 12.9)	5.6	(5.4; 5.8)	7.0	(6.7; 7.2)
LEL	11.0	(10.0; 12.0)	8.9	(7.7; 10.2)	7.7	(7.1; 8.3)	6.7	(5.9; 7.5)	4.7	(4.4; 5.0)	4.5	(4.1; 4.9)	2.2	(2.0; 2.4)	2.4	(2.2; 2.7)
PLEL (%)	34.9	(31.7; 38.2)	26.0	(22.4; 29.7)	34.1	(31.4; 36.7)	26.4	(23.3; 29.4)	32.0	(29.8; 34.2)	26.6	(24.1; 29.0)	27.8	(25.4; 30.2)	25.9	(23.4; 28.3)
**2018**																
LE	31.7		34.4		22.7		25.3		14.8		16.9		8.1		9.5	
LE CLL	23.5	(22.3; 24.7)	27.9	(26.5; 29.3)	16.9	(16.2; 17.6)	20.3	(19.5; 21.2)	11.2	(10.8; 11.6)	13.5	(13.0; 14.0)	6.4	(6.2; 6.6)	7.6	(7.3; 7.9)
LEL	8.2	(7.0; 9.4)	6.5	(5.1; 7.9)	5.8	(5.1; 6.6)	5.0	(4.1; 5.9)	3.6	(3.2; 4.0)	3.4	(2.9; 3.9)	1.7	(1.5; 1.9)	1.9	(1.6; 2.2)
PLEL (%)	25.9	(22.1; 29.8)	18.9	(14.8; 23.0)	25.6	(22.4; 28.9)	19.6	(16.1; 23.2)	24.2	(21.5; 26.9)	20.2	(17.3; 23.1)	21.0	(18.1; 23.8)	19.9	(17.0; 22.8)

As a reader's guide, a 50-year-old male in 1990 is estimated, on average, to have 30.3 life-years remaining. On the other hand, a 50-year-old male patient with CLL diagnosed in 1990 has, on average, 11.3 life-years remaining. Thus, a 50-year-old male patient with CLL loses, on average, 19 life-years due to a CLL diagnosis in 1990. This corresponds to a 50-year-old male patient with CLL losing, on average, 62.7% of his life due to CLL diagnosis in 1990. The 95% confidence intervals, shown in the Table in parentheses, are obtained using the Delta method. Abbreviations: CLL, chronic lymphocytic leukemia; LE, life expectancy; LEL, loss in expectation of life of CLL patients; PLEL, proportional loss in expectation of life of CLL patients.

Supplemental Table S3. The conditional loss in expectation of life of patients with chronic lymphocytic leukemia at diagnosis and after surviving five and ten years post-diagnosis. These survival measures, with associated 95% confidence intervals, are presented for four selected calendar years and ages at diagnosis.

	**Age 50 years**				**Age 60 years**				**Age 70 years**				**Age 80 years**			
**Measure**	**Males**		**Females**		**Males**		**Females**		**Males**		**Females**		**Males**		**Females**	
**1990**																
0-CLEL	19.0	(18.3; 19.8)	17.3	(16.2; 18.4)	11.7	(11.2; 12.1)	11.8	(11.1; 12.5)	6.1	(5.8; 6.4)	7.0	(6.7; 7.4)	3.1	(2.9; 3.3)	3.7	(3.5; 3.9)
5-CLEL	15.3	(14.4; 16.1)	14.6	(13.4; 15.8)	8.6	(8.1; 9.1)	9.3	(8.5; 10.0)	4.0	(3.7; 4.2)	4.8	(4.5; 5.2)	1.7	(1.5; 1.9)	2.1	(1.9; 2.3)
10-CLEL	11.1	(10.1; 12.1)	11.0	(9.7; 12.2)	5.6	(5.0; 6.1)	6.2	(5.5; 7.0)	2.2	(1.9; 2.5)	2.7	(2.4; 3.0)	0.8	(0.7; 1.0)	1.0	(0.8; 1.1)
**2000**																
0-CLEL	15.2	(14.5; 15.8)	12.8	(11.8; 13.8)	10.2	(9.9; 10.6)	9.3	(8.7; 9.9)	5.6	(5.4; 5.8)	5.9	(5.6; 6.2)	2.5	(2.3; 2.6)	3.0	(2.8; 3.1)
5-CLEL	12.2	(11.4; 13.0)	10.7	(9.7; 11.7)	7.7	(7.2; 8.1)	7.3	(6.7; 7.9)	3.8	(3.6; 4.1)	4.1	(3.8; 4.4)	1.5	(1.3; 1.6)	1.7	(1.6; 1.9)
10-CLEL	8.7	(7.8; 9.6)	8.0	(7.0; 9.0)	4.9	(4.4; 5.4)	4.8	(4.2; 5.4)	2.1	(1.9; 2.3)	2.2	(2.0; 2.5)	0.7	(0.6; 0.8)	0.8	(0.7; 0.9)
**2010**																
0-CLEL	11.0	(10.0; 12.0)	8.9	(7.7; 10.2)	7.7	(7.1; 8.3)	6.7	(5.9; 7.5)	4.7	(4.4; 5.0)	4.5	(4.1; 4.9)	2.2	(2.0; 2.4)	2.4	(2.2; 2.7)
5-CLEL	8.9	(7.9; 10.0)	7.5	(6.2; 8.7)	5.8	(5.2; 6.4)	5.2	(4.4; 6.0)	3.1	(2.8; 3.5)	3.1	(2.7; 3.5)	1.3	(1.1; 1.4)	1.4	(1.2; 1.5)
10-CLEL	6.3	(5.3; 7.3)	5.5	(4.4; 6.6)	3.7	(3.1; 4.2)	3.4	(2.8; 4.1)	1.6	(1.4; 1.9)	1.7	(1.4; 1.9)	0.6	(0.5; 0.7)	0.6	(0.5; 0.7)
**2018**																
0-CLEL	8.2	(7.0; 9.4)	6.5	(5.1; 7.9)	5.8	(5.1; 6.6)	5.0	(4.1; 5.9)	3.6	(3.2; 4.0)	3.4	(2.9; 3.9)	1.7	(1.5; 1.9)	1.9	(1.6; 2.2)
5-CLEL	6.7	(5.5; 7.9)	5.4	(4.1; 6.8)	4.4	(3.7; 5.1)	3.9	(3.0; 4.8)	2.4	(2.1; 2.8)	2.4	(1.9; 2.8)	1.0	(0.8; 1.2)	1.1	(0.9; 1.3)
10-CLEL	4.8	(3.7; 5.8)	4.0	(2.8; 5.2)	2.8	(2.2; 3.4)	2.6	(1.9; 3.3)	1.3	(1.0; 1.5)	1.3	(1.0; 1.6)	0.5	(0.3; 0.6)	0.5	(0.3; 0.6)

As a reader's guide, a 50-year-old male with CLL diagnosed in 1990 has, on average, 19.0 life-years remaining at diagnosis (0-CLEL), which turns to 11.1 life-years after surviving up to ten years post-diagnosis (i.e. 10-CLEL). The 95% confidence intervals, shown in the Table in parentheses, are obtained using the Delta method. Abbreviations: 0-CLEL, conditional loss in expectation of life at diagnosis; 5-CLEL, conditional loss in expectation of life after surviving five years post-diagnosis, 10-CLEL, conditional loss in expectation of life after surviving ten years post-diagnosis.

Supplemental Table S4. The proportional conditional loss in expectation of life of patients with chronic lymphocytic leukemia at diagnosis and after surviving five and ten years post-diagnosis. These survival measures, with associated 95% confidence intervals, are presented for four selected calendar years and ages at diagnosis.

	Age 50 years				Age 60 years				Age 70 years				Age 80 years			
**Measure (%)**	**Males**		**Females**		**Males**		**Females**		**Males**		**Females**		**Males**		**Females**	
**1990**																
0-PCLEL	62.7	(60.4; 65.1)	50.8	(47.5; 54.1)	58.7	(56.6; 60.9)	48.7	(45.9; 51.5)	53.3	(51.1; 55.5)	46.2	(43.8; 48.7)	48.7	(45.7; 51.7)	44.2	(41.3; 47.0)
5-PCLEL	58.6	(55.4; 61.9)	49.3	(45.2; 53.3)	52.8	(49.8; 55.7)	45.8	(42.3; 49.4)	44.8	(41.8; 47.8)	41.1	(38.0; 44.2)	36.7	(32.9; 40.4)	35.3	(31.8; 38.7)
10-PCLEL	50.3	(45.8; 54.9)	43.5	(38.6; 48.5)	42.4	(38.2; 46.6)	38.2	(33.8; 42.6)	32.8	(29.0; 36.6)	31.1	(27.5; 34.8)	24.6	(20.8; 28.5)	24.2	(20.6; 27.7)
**2000**																
0-PCLEL	48.5	(46.3; 50.7)	37.4	(34.6; 40.2)	46.5	(44.8; 48.3)	36.9	(34.6; 39.2)	42.6	(41.1; 44.2)	36.1	(34.3; 37.9)	36.8	(34.8; 38.8)	34.1	(32.2; 36.1)
5-PCLEL	45.3	(42.4; 48.2)	36.1	(32.7; 39.4)	42.1	(39.6; 44.5)	34.6	(31.7; 37.4)	36.8	(34.6; 39.0)	32.1	(29.8; 34.4)	29.4	(26.8; 32.1)	27.7	(25.3; 30.1)
10-PCLEL	38.4	(34.5; 42.3)	31.4	(27.5; 35.4)	33.5	(30.0; 36.9)	28.4	(24.9; 31.8)	26.8	(23.9; 29.7)	23.9	(21.1; 26.7)	19.8	(17.1; 22.6)	18.6	(16.1; 21.0)
**2010**																
0-PCLEL	34.9	(31.7; 38.2)	26.0	(22.4; 29.7)	34.1	(31.4; 36.7)	26.4	(23.3; 29.4)	32.0	(29.8; 34.2)	26.6	(24.1; 29.0)	27.8	(25.4; 30.2)	25.9	(23.4; 28.3)
5-PCLEL	32.9	(29.0; 36.8)	25.1	(20.9; 29.3)	31.2	(27.9; 34.5)	24.8	(21.1; 28.4)	28.0	(25.3; 30.8)	23.7	(20.7; 26.6)	22.9	(19.9; 25.8)	20.9	(18.1; 23.8)
10-PCLEL	27.8	(23.4; 32.3)	21.7	(17.2; 26.2)	24.8	(21.1; 28.6)	20.2	(16.3; 24.1)	20.3	(17.3; 23.3)	17.5	(14.4; 20.5)	15.4	(12.6; 18.1)	13.8	(11.2; 16.4)
**2018**																
0-PCLEL	25.9	(22.1; 29.8)	18.9	(14.8; 23.0)	25.6	(22.4; 28.9)	19.6	(16.1; 23.2)	24.2	(21.5; 26.9)	20.2	(17.3; 23.1)	21.0	(18.1; 23.8)	19.9	(17.0; 22.8)
5-PCLEL	24.6	(20.2; 29.1)	18.3	(13.7; 22.9)	23.8	(20.0; 27.7)	18.5	(14.5; 22.6)	21.7	(18.5; 24.9)	18.1	(14.8; 21.5)	17.8	(14.5; 21.1)	16.3	(13.1; 19.6)
10-PCLEL	20.9	(16.3; 25.6)	15.8	(11.1; 20.5)	19.1	(15.1; 23.1)	15.1	(11.0; 19.2)	15.9	(12.7; 19.1)	13.4	(10.2; 16.6)	12.2	(9.3; 15.2)	10.8	(8.0; 13.6)

As a reader's guide, a 50-year-old male with CLL diagnosed in 1990 loses, on average, 62.7% of their life expectancy (0-PCLEL), which turns to 50.3% after surviving up to ten years post-diagnosis (10-PCLEL). The 95% confidence intervals, shown in the Table in parentheses, are obtained using the Delta method.
Abbreviations: 0-PCLEL, proportional conditional loss in expectation of life at diagnosis; 5-PCLEL, proportional conditional loss in expectation of life after surviving five years post-diagnosis; 10-PCLEL, proportional conditional loss in expectation of life after surviving ten years post-diagnosis.

Supplemental figure legends

Supplemental Figure 1. The proportional conditional loss in expectation of life of patients with chronic lymphocytic leukemia diagnosed in the Netherlands. The proportional conditional loss in expectation of life is presented according to four age groups at diagnosis, stratified by sex and four selected calendar periods of diagnosis. The projected measures of life expectancy according to selected years of diagnosis are presented in Supplemental Table 4.

Supplemental Figure 1

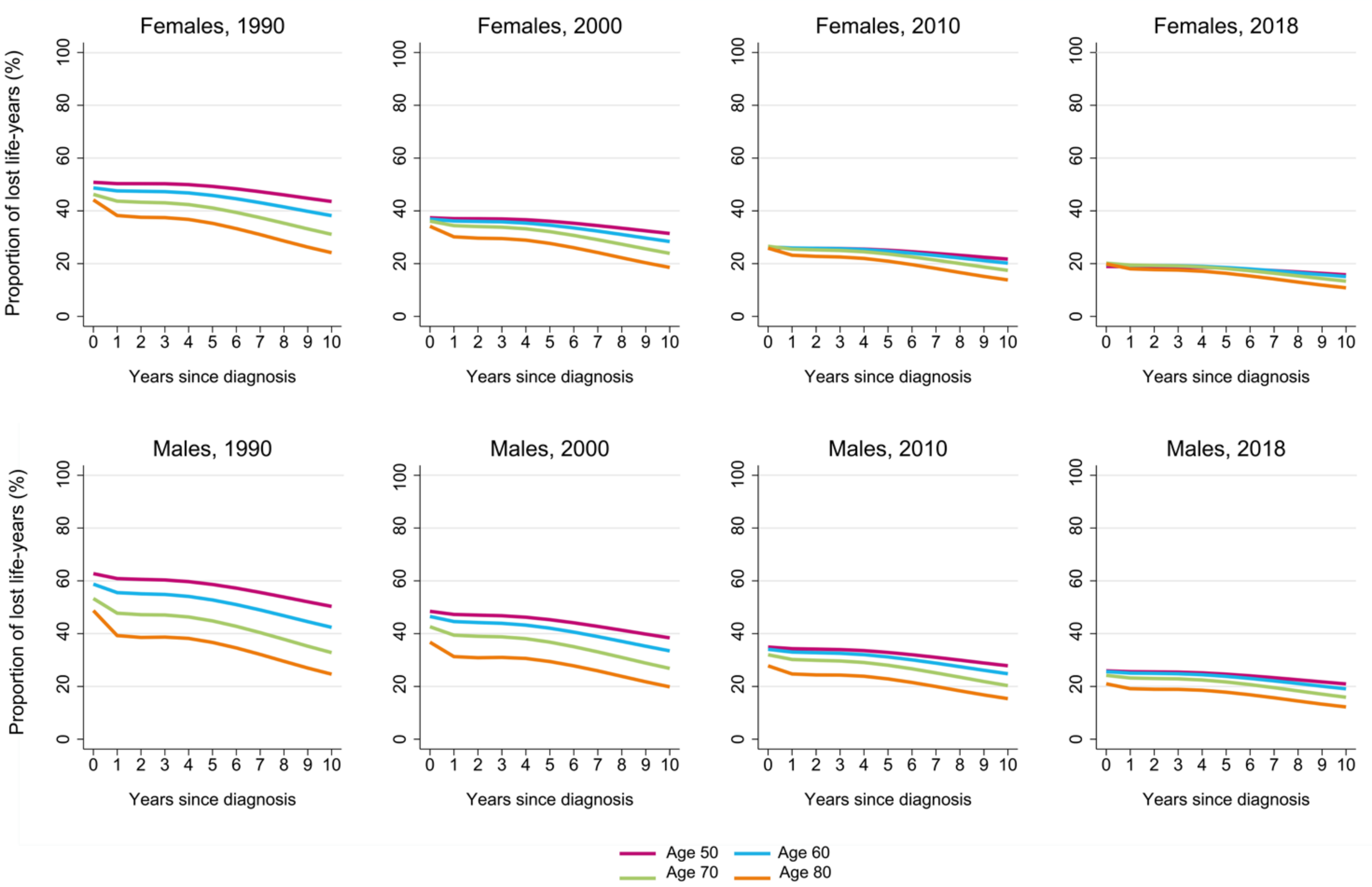

Supplemental references

1. Maas CCHM, van Klaveren D, Ector GICG, Posthuma FM, Visser O, Westerweel PE, et al. The evolution of the loss of life expectancy in patients with chronic myeloid leukaemia: a population-based study in the Netherlands, 1989-2018. Br J Haematol. 2021.

2. van der Straten L, Levin M-D, Visser O, Posthuma EFM, Doorduijn JK, Kater AP, et al. Conditional relative survival among patients with chronic lymphocytic leukaemia: A population-based study in the Netherlands. eJHaem. 2022;3(1):180-3.
